# First New Zealand fur seal population assessment at the Bounty Islands in 30 years

**DOI:** 10.7717/peerj.20975

**Published:** 2026-03-27

**Authors:** Alasdair A. Hall, Thomas Mattern, Kalinka Rexer-Huber, Klemens Pütz, Jody S. Weir

**Affiliations:** 1 Hall Marine Consulting, Wellington, Wellington, New Zealand; 2 Department of Zoology, University of Otago, Dunedin, Otago, New Zealand; 3 The Tawaki Trust, Dunedin, Otago, New Zealand; 4 Global Penguin Society, Puerto Madryn, Patagonia, Argentina; 5 Parker Conservation, Dunedin, Otago, New Zealand; 6 Antarctic Research Trust, Bremervörde, Germany; 7 Department of Conservation, Kaikōura, New Zealand

**Keywords:** *Arctocephalus forsteri*, Sub-Antarctic, Population monitoring, Unmanned aerial vehicle, Bounty Islands, New Zealand fur seal

## Abstract

A common pitfall in wildlife population monitoring is targeting effort towards locations which are easily accessible due to logistical and resource constraints. This can limit nuanced understandings of population dynamics for species which experience varying stressors through space. The only long-term monitoring programmes for New Zealand fur seal (*Arctocephalus forsteri*) in New Zealand have occurred within central areas of their nationwide range, and colonies in the sub-Antarctic have been particularly neglected. The last population assessment for New Zealand fur seal on the Bounty Islands was in 1994, when ∼21,500 animals were estimated. This population suffers high by-catch rates in commercial fisheries and may be at elevated risk of High Pathogenicity Avian Influenza due to cohabitation with wide-ranging pelagic seabirds. This study uses imagery collected by an unmanned-aerial-vehicle (UAV) to estimate pup abundance in the 2023/24 breeding season from the Main Group of the Bounty Islands. A total of 4,168–4,256 pups (mean ± SD) were manually counted from high resolution orthomosaics, with pups recorded on eight of the 13 islands/rocks assessed. After accounting for pups that may have been obscured from the UAV images, two established population multipliers were adopted to convert pup production estimates into rough total population estimates for the islands assessed, giving estimates of 20,237–20,663 and 20,832–21,271 respectively. While these results are similar to the 1994 estimate, direct comparisons are constrained by differences in methodologies and survey timing. In addition to providing an important new baseline for an understudied New Zealand fur seal population facing multiple threats, this research demonstrates the benefits of UAVs for population monitoring in regions where traditional methods cannot be easily implemented.

## Introduction

Understanding the contemporary abundance and distribution of wildlife populations is important for management and conservation (Ransom et al., 2012; Wege et al., 2016; Forsyth et al., 2022). However, collecting population level data is often time and resource intensive (Wittmer, Sinclair & McLellan, 2005; Prosekov et al., 2020), particularly for populations in difficult to access locations (Shah et al., 2020). This can engender patchy and inconsistent coverage of wildlife abundance (Clayton & Cowan, 2010; Bruce et al., 2025), with study site selection sometimes biased by accessibility or proximity (Mentges et al., 2021; Arazy et al., 2024). If select monitored populations are used as proxies for the wider meta-population, this can mask nuances among any populations experiencing divergent trajectories due to localised stressors (Kirkman et al., 2011; Wall et al., 2023).

New Zealand fur seal/kekeno (*Arctocephalus forsteri*) are one of nine species within the *Arctocephalus* genus (Chilvers, 2018). The northernmost extent of the species’ contemporary breeding range is West Australia (Campbell et al., 2014), while the southernmost is on Macquarie Island (Chilvers & Harcourt, 2021). Prior to anthropogenic exploitation, there may have been as many as three million New Zealand fur seals in New Zealand, with a combination of subsistence and commercial hunting thought to have reduced the population size to less than 1% of this figure (Smith, 1996; Emami-Khoyi et al., 2018). New Zealand’s subantarctic region likely served as a partial refuge from hunting for New Zealand’s pinnipeds (Wing et al., 2025). Since the cessation of sealing, New Zealand fur seal populations have increased in several parts of New Zealand (Dix, 1993; Lalas & Harcourt, 1995; Boren, Muller & Gemmell, 2006; Watson, Beaven & Bradshaw, 2015; Chilvers, 2021; Hall, Chilvers & Weir, 2025a), with some populations thought to have stabilised (Carey, 1998; MacDiarmid & Lalas, 2014). However, three colonies on the West Coast of the South Island (WCSI) have experienced substantial declines in pup production and condition over the last ∼30 years (Hall et al., 2026). The most recent nationwide abundance estimate for New Zealand fur seal in New Zealand, based on modelling, is approximately 181,646–239,473 (Hall, Chilvers & Weir, 2025b).

New Zealand’s New Zealand fur seals have been inconsistently monitored since the species began to recover from sealing (Lalas & Bradshaw, 2001; Hall, Chilvers & Weir, 2025b). Colonies on New Zealand’s subantarctic islands have been particularly neglected (Hall, Chilvers & Weir, 2025b) with some, such as the Auckland Islands, never surveyed, and most others last surveyed in 1980s or 1990s (Moore & Moffat, 1990; Taylor, 1992; Taylor, 1996; Carey, 1998). Mattern (2024) provided an estimate of 940 New Zealand fur seals at Reef Point on Antipodes Island in January 2024 using pup counts from unmanned-aerial-vehicle (UAV) surveys and a population multiplier (Taylor, 1996). The last New Zealand fur seal abundance assessment from the Bounty Islands/Moutere Hauriri (‘Bounties’) was conducted in the 1993/1994 breeding season (hereafter, breeding seasons are referred to by the later year included, *i.e.,* 1994) when 4,380 pups were estimated from photographs taken from an aeroplane in January 1994 (Taylor, 1996). This pup estimate was then subjected to a multiplier of 4.9 (Taylor, 1996) to produce a population estimate of 21,500 (Taylor, 1996). There are several potential flaws in how Taylor (1996) arrived at his pup and, therefore, population estimates which complicate direct comparisons with contemporary methodologies (see Discussion).

Monitoring subantarctic New Zealand fur seal colonies is important for several reasons. First, it has been suggested that high pathogenicity avian influenza (HPAI) may reach New Zealand *via* the Southern Ocean (Gartrell, Jolly & Hunter, 2024). The virus has had devastating impacts on pinniped populations in other countries (Ulloa et al., 2023; Leguia et al., 2023; Campagna et al., 2024) and, given that pinnipeds can act as sentinels for disease (Baily et al., 2016) monitoring subantarctic New Zealand fur seal populations may reveal the presence of pathogens like HPAI before they reach mainland New Zealand. In addition, commercial fisheries operate around New Zealand’s subantarctic islands, with bycatch thought to be at least partially responsible for the decline in New Zealand sea lion (*Phocarctos hookeri*) abundance on the Auckland Islands (Meyer et al., 2017). There are high rates of New Zealand fur seal bycatch around the Bounties, especially in the southern blue whiting fishery (*Micromesistius australis*) (Taylor, 1996; Abraham, Tremblay-Boyer & Berkenbusch, 2021). However, the lack of New Zealand fur seal population data means the impacts of both bycatch and prey depletion are not well understood. Finally, given the ecological distinctiveness of the subantarctic (Selkirk, 2007), it should not be assumed that New Zealand fur seal colonies there have experienced similar trends to those on mainland New Zealand.

The advent and improvement of unmanned aerial vehicle (UAV) technology has facilitated assessments of wildlife populations in difficult to access locations—such as Adélie penguin (*Pygoscelis adeliae*) colonies in Antarctica (Shah et al., 2020), European elk (*Alces alces*) in the Salair Mountains (Prosekov et al., 2022), wild yaks (*Bos mutus*) in the Kumkury Desert (Su et al., 2018) and cliff-nesting Eleonora’s falcons (*Falco eleonorae*) in Cyprus (Hadjikyriakou et al., 2020). Additionally, UAV surveys typically require fewer personnel and resources, as well as less time in the field, when compared to traditional abundance assessment methods (Sardà-Palomera et al., 2012; Anderson & Gaston, 2013; Sorrell et al., 2019). UAVs have been used successfully to assess pinniped population sizes (McIntosh, Holmberg & Dann, 2018; Sorrell et al., 2019; Infantes et al., 2022), and have the added benefit of reducing disturbance compared to common monitoring approaches such as ground counts or mark-recapture (Sorrell et al., 2019). In October 2019, Rexer-Huber & Parker (2020) conducted feasibility trials for using UAVs to count New Zealand fur seals on the Bounties. They tested the responses of wildlife present on the Bounties (New Zealand fur seal, penguins and seabirds) to UAV flights and trialled the flight parameters necessary to collect high quality images for estimating New Zealand fur seal abundance. They found flights at 40 m, 60 m or 80 m above the launch site only elicited reactions from gulls and hovering above 20 metres was largely ignored by New Zealand fur seals (Rexer-Huber & Parker, 2020). Excellent quality imagery suitable for New Zealand fur seal abundance monitoring was obtained from flights at 40 m and 60 m. From Proclamation Island, a total of 1,154 New Zealand fur seals were counted, including 341 ∼10 month-old pups (Rexer-Huber & Parker, 2020). The October timing of Rexer-Huber & Parker’s (2020) survey means these pup counts cannot be used to estimate pup production, as by this time pups have moulted into their adult pelage and cannot be distinguished with certainty. However, the feasibility study demonstrated the viability of counting New Zealand fur seal pups using UAV imagery at the Bounties. Typically, New Zealand fur seal pupping on mainland New Zealand occurs between late November and early January (Chilvers, 2021). Therefore, surveys usually occur between mid-January—early March, coinciding with a period where all pups have been born but still retain their unique black natal pelage, when most territorial breeding males have departed the colony and before pups are competent swimmers and able to spend significant time in the ocean (Boren, Muller & Gemmell, 2006; Chilvers, 2021).

In this study, we use UAV images collected in January 2024 to provide a new baseline estimate for the number of New Zealand fur seal pups produced in the Main Group of the Bounties, for the first time in 30 years (Taylor, 1996). This is also the first robust New Zealand fur seal population assessment from any of New Zealand’s subantarctic islands since an assessment of the Snares’ population in 1997 (Carey, 1998).

The objectives of this study were:

(i) To provide a pup production estimate for the Main Group of islands within the Bounties archipelago in 2024.
(ii) From this, to produce a partial population estimate for the Bounties from the areas surveyed.
(iii) Based on our findings, to make recommendations for future usage of UAVs to monitor New Zealand fur seals at the Bounties and elsewhere in New Zealand.

## Methods

### Study site

**Figure 1:**
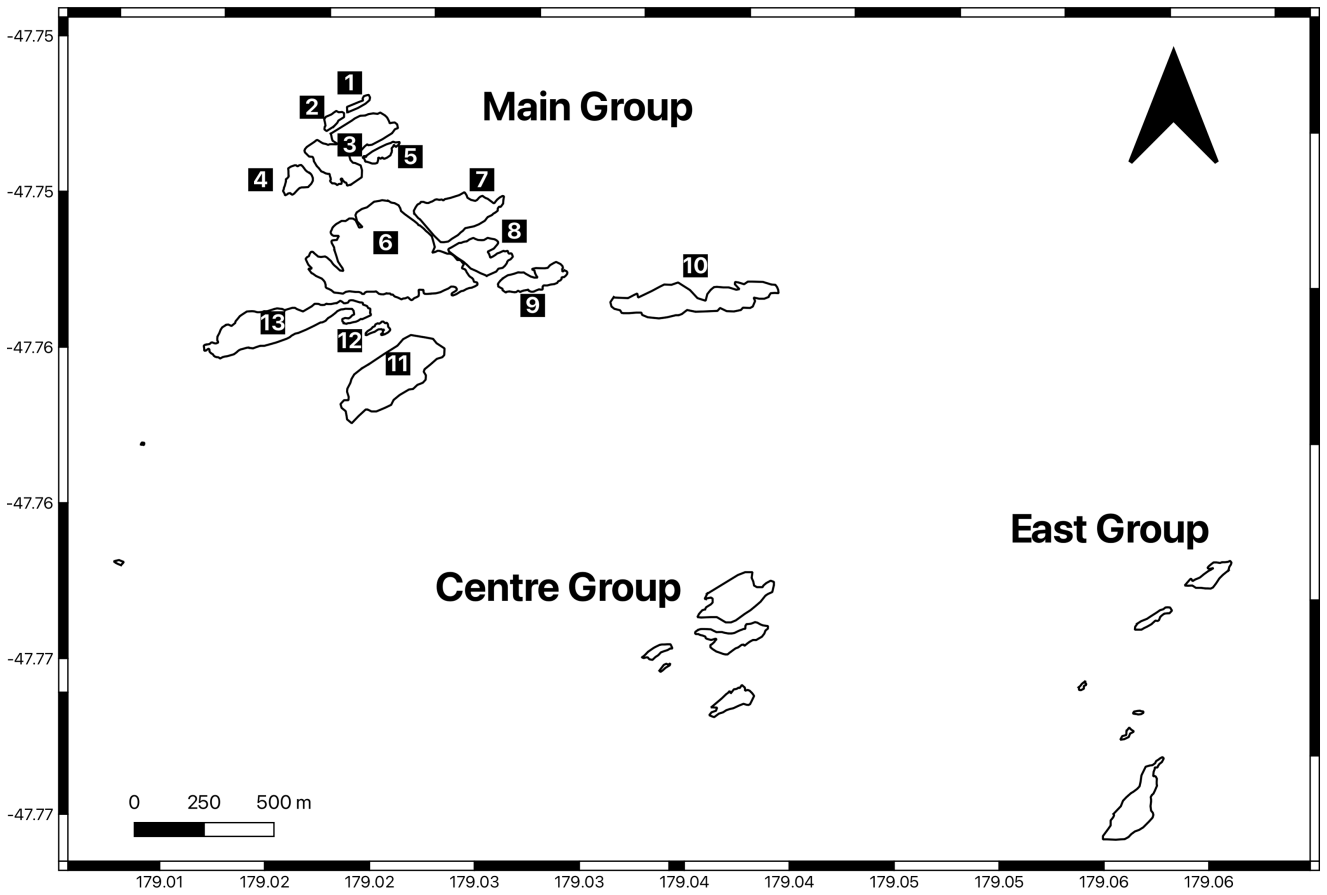
The Bounty Islands archipelago. 1. Unnamed Rock A, 2. Unnamed Rock B, 3. Spider Island, 4. Skua Rock, 5. Seal Rock, 6. Depot Island, 7. Proclamation Island, 8. Tunnel Island, 9. Ranfurly Island, 10. Lion Island, 11. Ruatara Island, 12. Dog Rock, 13. Penguin Island. Image created in QGIS.

The Bounties (Fig. 1) are a collection of 14 islands and 11 rocks, ranging in size from 0.4 to 12.7 ha. The islands and rocks are barren and granite, and rise to heights of 40–60 metres above sea level (Mattern et al., 2023). The Bounties are uninhabited, and typical environmental conditions include strong winds, frequent rainfall and cloud.

In addition to New Zealand fur seals, the Bounties are home to Salvin’s albatross (*Thalassarche salvini*) (Sagar et al., 2015), erect-crested penguins (*Eudyptes sclateri)* (Wilson & Mattern, 2019)*,* as well as fulmar prions (*Pachyptila crassirostris*) (Shepherd et al., 2022) and the endemic Bounty Island shag (*Leucocarbo ranfurlyi*) (Mattern et al., 2023).

The Bounties archipelago is divided into three groups of islands and rocks (Fig. 1): Main Group, Centre Group and East Group. This study covered all the islands and rocks in the Main Group, which has historically been where the majority of New Zealand fur seals have been observed (Taylor, 1982; Taylor, 1996).

### Abundance estimates in pinnipeds

Pinniped abundance estimates are typically conducted through pup counts, as this cohort provides the best index for the total population size (Berkson & De Master, 1985; Chilvers & Goldsworthy, 2015; Chilvers, 2021; Hammond et al., 2021; McIntosh et al., 2022; Hall, Chilvers & Weir, 2025a). Unlike weaned individuals, an unknown proportion of which may be at sea when population assessments are conducted, pre-weaned New Zealand fur seal pups (aged up to 9–10 months; Boren, 2005) are largely restricted to their breeding colony before they moult (usually at 3–4 months old) as their natal pelage is not waterproof (Chilvers & Goldsworthy, 2015). Additionally, the black colouration of this natal pelage means pups can be easily differentiated from older individuals (Boren, Muller & Gemmell, 2006; Chilvers, 2021). Consistently executed pup counts can, therefore, be used to track population trajectories (Chilvers, Wilkinson & Childerhouse, 2007; McIntosh et al., 2022; Hall et al., 2026).

Approximate population sizes can be derived by subjecting pup production estimates to multipliers (Shaughnessy, Goldsworthy & Libke, 1995; Chilvers, 2021; Hall et al., 2024; Hall, Chilvers & Weir, 2025a). In this study, two multipliers were adopted. The first (4.76), calculated by Goldsworthy & Page (2007), was included to permit comparisons between the Bounties’ results and recent population studies from mainland New Zealand (Chilvers, 2021; Hall et al., 2024; Hall, Chilvers & Weir, 2025a). Goldsworthy & Page’s (2007) multiplier was derived from lifetables of New Zealand fur seals in Australia, found in the paper’s Appendix A. The second (4.9) was previously used by Taylor (1982) and Taylor (1996) to estimate the size of the Bounties’ New Zealand fur seal population and was used for historical comparisons. This multiplier was based on vital rates estimated from other *Arctocephalus* species (Taylor, 1996). Both multipliers have been previously used to convert pup production estimates into population estimates (Shaughnessy, Goldsworthy & Libke, 1995; Shaughnessy, Stirling & Dennis, 1996; Chilvers, 2021; Hall et al., 2024; Hall, Chilvers & Weir, 2025a), however their results should be treated as coarse estimates given the limitations of such multipliers (see Discussion).

### UAV surveys

This study adheres to the relevant national, international (Field et al., 2019) and institutional guidelines for animal care. A total of 4,089 images covering the landmass within the Main Group of the Bounties were captured from UAV flights on January 31st, 2024 as part of the Tawaki Project (http://dx.doi.org/10.13140/RG.2.2.32269.14568) to monitor erect-crested penguins. Animal care approval for this work was granted by the University of Otago Animal Ethics Committee (University of Otago Animal Use Protocol number: AUP D69/17), and a research permit was granted by the New Zealand Department of Conservation (Wildlife Authorisation number: 86101-FAU) in accordance with the Marine Mammals Protection Act 1978 and Marine Mammals Protection Regulations 1992. The research was commended by the Ngāi Tahu Research Consultation Committee (Te Komiti Rakahau Ki Tahu).

Imagery was collected using a DJI Mavic 2 Pro UAV (Shenzhen DJI Sciences and Technologies, Nanshan, Shenzen, China). This UAV has a L1D-20c Hasselblad camera featuring a fixed 35 mm lens with an approximate field of view (FOV) of 77° which can capture still images at a resolution of 5,473 × 3,648 pixels (20 Megapixels). For each survey, the UAV was launched from the top of Proclamation Island (Fig. 1). DroneDeploy (https://www.dronedeploy.com) software was used to plan flights, which permits autonomous UAV flight along predetermined paths. Flight altitudes were selected so that the UAV would survey from 40–60 metres above each island’s highest point. The feasibility study (Rexer-Huber & Parker, 2020) demonstrated that flights between 40–60 metres produced high quality imagery for New Zealand fur seal counts on the Bounties, while not noticeably impacting animals on the ground. Similar altitudes have been used for surveying Australian fur seals (*Arctocephalus pusillus doriferus*) (McIntosh, Holmberg & Dann, 2018). Nadir images were taken with 80% front and 72% side overlap. This was to allow for the fact that the steep sides of the Bounties would mean greater distance to the ground along the coasts than further inland (Rexer-Huber & Parker, 2020). During all flights, a dedicated spotter maintained direct line of sight with the UAV, with binoculars used for flights flown further from the launch site.

### Image processing and pup counts

Images captured from the UAVs were used to produce georeferenced and textured 3D models of the islands. Images were processed using a structure-from-motion photogrammetric workflow in DroneDeploy (https://www.dronedeploy.com), to generate georeferenced orthomosaics (Mattern et al., 2023). This approach differs from simple image stitching in that surface reconstruction is based on the identification of features that are spatially consistent across multiple overlapping images. Consequently, static landscape features are reinforced during reconstruction, whereas objects that change position between successive image acquisitions are less consistently represented in the final orthomosaic. As a result, potential “ghosting” arising from pup movement between adjacent transects is expected to be reduced relative to non-photogrammetric mosaicking approaches. While some movement-related uncertainty is unavoidable, the combination of high image overlap, short temporal separation between transects, and limited pup mobility is expected to minimise the likelihood that individuals are duplicated as distinct features in the orthomosaic.

**Figure 2:**
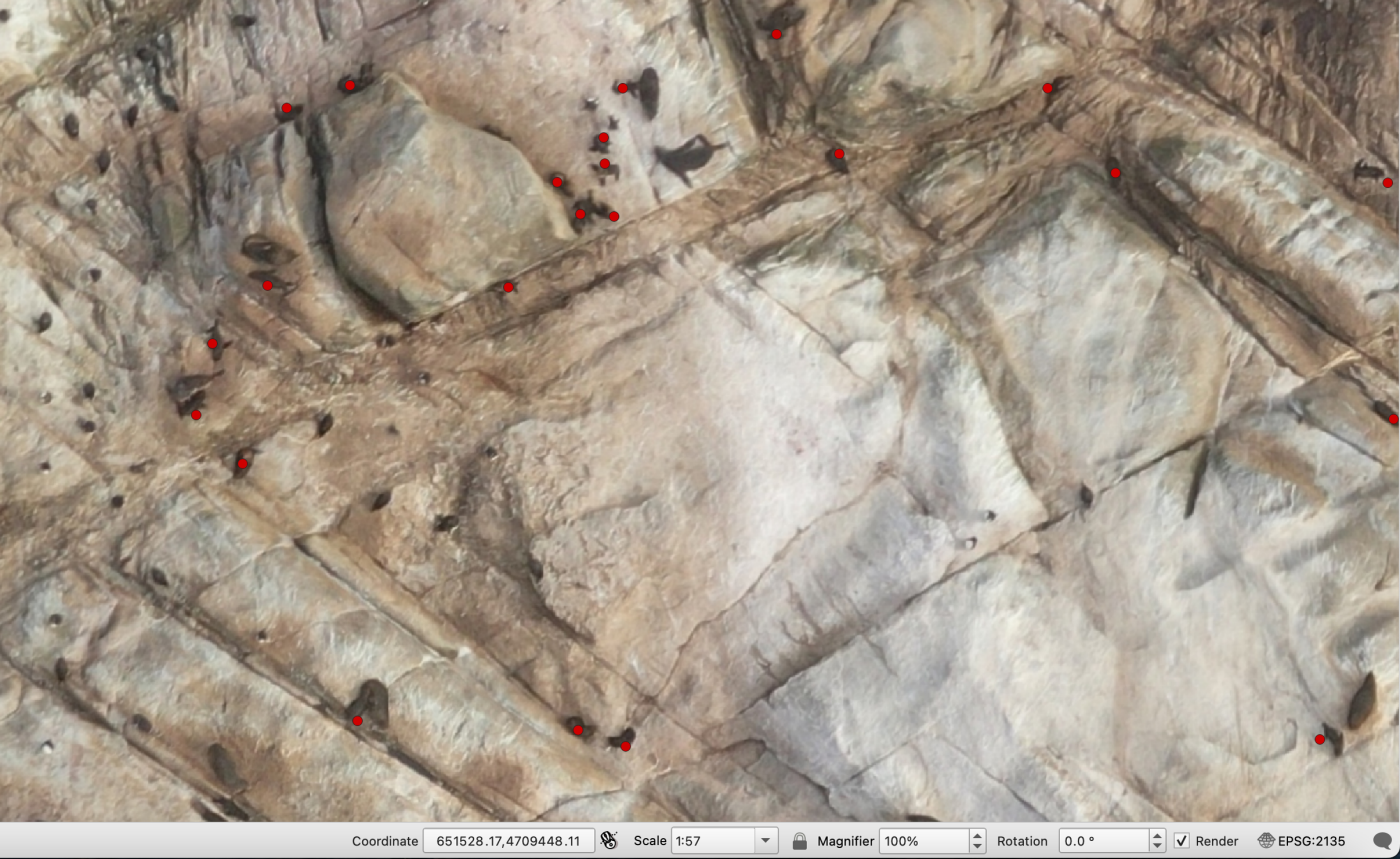
Screenshot of pup counts in QGIS from Depot Island. New Zealand fur seal pups are marked with red dots.

The orthomosaics were uploaded into QGIS (vers. 3.42.2) (QGIS Development Team, 2025) for pup counting (Fig. 2). A grid overlay was added to the images to facilitate navigation while zoomed, and pups were marked by creating a Point Shapefile layer and clicking on a pup to count it. The same analyst conducted all counts, and the results of two counts from each island/rock were averaged to provide results and standard deviations.

Pups were distinguished from other cohorts of New Zealand fur seals, as well as bird species present on the Bounties through a combination of colour, shape, size and behaviour.

As well as having a black natal pelage, which contrasts with the brown/silver colour of adults, New Zealand fur seal pups at this age are considerably smaller and have a narrower girth than adults. It was common to see pups suckling adult females, which not only provided a certain identification for those individuals, but also provided a good relative size comparison.

Body shape, size and colouration were used to distinguish pups from birds. (1) Body shape/size—when standing or moving, the presence of flippers on the pups contrasted with the smooth, tapered-oval shapes of Salvin’s albatross, fulmar prions, erect-crested penguins and Bounty Island shags when sitting with their wings tucked in. Erect-crested penguins, when standing upright and viewed from above, have a much shorter visible dorsal body length than New Zealand fur seal pups, while the slender body shape of fulmar prions and Bounty Island shags contrasts with the more tubular body of New Zealand fur seal pups, even if the pups’ flippers were not visible. (2) Colour—when viewed from above, the white head of the Salvin’s albatross is visible, as, sometimes, is the white breast of the erect-crested penguin, while blue/grey feathers of the fulmar prion are lighter than the black natal pelage of New Zealand fur seal pups. If, after consideration of these factors, a positive identification could not be made with certainty, an animal was not included in the pup count.

### Bias considerations for UAV pup counts

McIntosh, Holmberg & Dann (2018) describe three main types of biases that should be considered during count surveys, including UAV surveys

(1) Availability bias: all individuals should be equally observable. In our surveys, some pups will have been obscured from view, for example by being hidden under rocks. Taylor (1996) previously estimated that less than 2% of New Zealand fur seals will be obscured from aerial images taken on the Bounties. So, 2% was added to the pup counts obtained to account for this.
(2) Observer/counter bias: different counters will count different numbers of animals. This was minimised in this instance by using a single counter, who conducted multiple counts to provide an indication of error.
(3) Movement bias: pups could be double-counted if they move between locations during the survey. While this cannot be entirely mitigated, the flight parameters selected and process for orthomosaic creation (detailed above) should reduce the likelihood of individuals being duplicated across images.

## Results

Breeding, evidenced by the presence of New Zealand fur seal pups, was recorded on Spider Island, Seal Rock, Depot Island, Proclamation Island, Penguin Island, Ranfurly Island, Ruatara Island and Lion Island (Fig. 1). The distribution of pups on these islands can be found in the images in the Supplementary Information. No pups were recorded on Tunnel Island, Dog Rock, Skua Rock, Unnamed Rock A or Unnamed Rock B (Fig. 1).

Pup counts for each surveyed island or rock are presented in Table 1.

**Table 1:** New Zealand fur seal pup counts from UAV surveys at the Bounty Islands in January 2024.

Island/Rock	Mean pup count (± SD) from Jan 31, 2024
Unnamed Rock A	0
Unnamed Rock B	0
Spider Island	231 (± 5)
Seal Rock	112 (± 2)
Skua Rock	0
Depot Island	1,910 (±10)
Proclamation Island	491 (± 1)
Tunnel Island	0
Ranfurly Island	308 (± 6)
Penguin Island	809 (± 6)
Dog Rock	0
Ruatara Island	228 (± 7)
Lion Island	125 (± 6)
Pup total	4,168–4,256^*^
Pup total +2%	4,252–4,341^*^
Partial population estimate 1 (pup total +2% × 4.76^1^)	20,237–20,663^*^
Partial population estimate 2 (pup total + 2% × 4.9^2^)	20,832–21,271^*^

**Notes.**

1 Goldsworthy & Page (2007).

2 Taylor (1982).

* Range indicative of mean ± SD.

Of the surveyed islands, the largest, Depot Island, had the highest pup count (1,910 ± 10 SD), followed by Penguin Island (809 ± 6 SD) and then Proclamation Island (491 ± 1 SD). After adding 2% to account for pups that may have been obscured from the UAV’s perspective, the total number of pups on the islands surveyed was estimated at 4,252–4,341 (mean ± SD).

When this total was subjected to the population multipliers of Goldsworthy & Page (2007) and Taylor (1982) respectively, population estimates of 20,237–20,663 and 20,832–21,271 New Zealand fur seals were estimated for the surveyed islands within the Bounties archipelago in January 2024.

## Discussion

This study provides the first comprehensive data on New Zealand fur seal breeding at the Main Group of the Bounties in 30 years (Taylor, 1996), and the first from any of New Zealand’s subantarctic islands since Carey’s 1997 (1998) assessment of the Snares Islands. While these results provide a useful baseline for future New Zealand fur seal monitoring at the Bounties, it should be remembered that this survey did not cover the entire archipelago (although previous surveys suggest that only a small proportion of the Bounties’ New Zealand fur seal population inhabit the unsurveyed islands, see ‘Study limitations’). Therefore, it is likely that the estimate of ca. 20,000–21,000 New Zealand fur seals in January 2024 (Table 1) underestimates the true population size. New Zealand fur seal counts from recent UAV surveys of the Bounties’, such as those derived from the feasibility study in October 2019 (Rexer-Huber & Parker, 2020) and a subsequent survey in November 2022 (Mattern, 2022) should not be interpreted in relation to the results produced in this study due to their timing prior to the commencement of pupping.

The closest known New Zealand fur seal breeding colonies to the Bounties are the Antipodes Islands (Taylor, 1992), located approximately 215 km to the south. Given the distances New Zealand fur seals are known to travel (Page et al., 2006), it is likely that there is some migration in and out of the Bounties’ population, including potentially to mainland New Zealand (∼700 km away). However, given the high degree of natal site fidelity among female New Zealand fur seals (Stirling, 1971; Chilvers, 2018) and the Bounties’ remoteness, it can be assumed that the Bounties’ breeding population is largely isolated.

### Conservation and management implications

The abundance estimates derived from this study provide an important new baseline for the population of New Zealand fur seals on the Bounties, which faces threats including high levels of bycatch (Taylor, 1996; Abraham, Tremblay-Boyer & Berkenbusch, 2021), the presence of HPAI in the subantarctic region (Bennison et al., 2024) and climate induced changes to their environment (Muller et al., 2024; Lattuca et al., 2025). At the time of writing, the HPAI virus subtype (H5N1) has never been detected in New Zealand, however, there are serious concerns about the impacts of an outbreak on New Zealand’s wild birds and commercial poultry flocks (Stanislawek et al., 2024; Waller et al., 2025). Particularly high-risk periods include September and November, when large numbers of migratory birds return to New Zealand (Williams, 2006). Evidence from overseas suggests that New Zealand’s pinnipeds may be at substantial risk from HPAI (Ulloa et al., 2023; Leguia et al., 2023; Campagna et al., 2025). In 2023, southern elephant seal (*Mirounga leonina*) pup mortality at a beach in Península Valdés, Argentina, reached 96% by the end of the breeding season (Campagna et al., 2024), while at least 24,000 South American sea lions (*Otaria flavescens*) died from HPAI in Chile, Peru, Brazil, Argentina and Uruguay between January–October 2023 (Plaza et al., 2024). The most likely infection routes were thought to be consuming or cohabiting with infected birds (Plaza et al., 2024). As HPAI has already been detected elsewhere in the subantarctic (Bennison et al., 2024), there are concerns that it will become established among Southern Ocean seabirds and reach mainland New Zealand from the south (Gartrell, Jolly & Hunter, 2024). If HPAI reaches New Zealand’s subantarctic, monitoring populations of New Zealand fur seals would not only enable understandings of the virus’ impacts on this ecologically important top predator, but may also serve as an early warning of its proximity to mainland New Zealand (Bossart, 2011; Núñez Egido et al., 2020), and to endangered New Zealand sea lions (*Phocarctos hookeri*) in the subantarctic. To achieve this, regular New Zealand fur seal monitoring at the Bounties, as well as other subantarctic islands, would be required so that mortality and population size can be compared before and after the virus’ arrival (Timm et al., 2009; Campagna et al., 2024). The ability of such monitoring to detect the impacts of a pathogen on a New Zealand fur seal population was recently demonstrated in Kaikōura, where pup production halved and dead pup counts tripled at Ōhau Point between two consecutive breeding seasons (A. Hall, B.L. Chivers, L. Angus, O. Gooday, J. Weir, 2024–2025, unpublished data/in prep), with necropsied pups testing positive for a divergent strain of canine distemper virus (Wilson et al., 2025).

The Bounties area also has a high rate of New Zealand fur seal bycatch, particularly in the southern blue whiting fishery (Abraham, Tremblay-Boyer & Berkenbusch, 2021). Between the October 2019/20 and September 2024/25 fishing years, there were 69 incidences of reported New Zealand fur seal bycatch in the New Zealand’s subantarctic commercial fishing area (FMA 6), 64 of which were fatal (Ministry for Primary Industries of New Zealand, 2025). Limited understandings of New Zealand fur seal population sizes and trajectories throughout much of New Zealand currently inhibit interpretation of the impacts of bycatch on specific New Zealand fur seal colonies or populations (Abraham et al., 2017). However, high rates of bycatch on the WCSI could be associated with long-term declines in New Zealand fur seal abundance at three colonies (Hall et al., 2026).

Climate change is another threat, and can impact pinnipeds in various ways both on land and at sea (Kovacs et al., 2012). Among these are alterations to the availability of prey species due to changing marine environmental conditions (Kliska et al., 2022; Bas et al., 2025; Guerreiro et al., 2025). Variable food availability impacts the ability of female pinnipeds to provision their pups (Cruz-Vallejo et al., 2024), which may impact pup survival and thus future population size (Beauplet et al., 2005; Wall et al., 2023). Climate induced changes to the marine ecosystem could be at least partially responsible for the declines in New Zealand fur seal pup abundance at three study colonies on the WCSI (Hall et al., 2026). Therefore, consistently executed monitoring may be able to detect the impacts of climate change on New Zealand fur seals in the subantarctic. However, it is likely that foraging behaviour and diet studies would also be required to confidently define the drivers of any detected population change (Speakman et al., 2020; Speakman, McHuron & Arnould, 2024; Cruz-Vallejo et al., 2024).

This study adds to the growing body of work demonstrating the advantages of using UAVs to monitor pinniped populations for conservation and management (McIntosh, Holmberg & Dann, 2018; Sorrell et al., 2019; Larsen et al., 2022; Hawkins et al., 2023), and demonstrates how UAVs can enable monitoring of multiple species from a single set of images. For remote and inaccessible locations such as the Bounties, UAV technology provides an efficient way to monitor New Zealand fur seal populations which would otherwise likely go unassessed. The process could be further expedited in the future through the use of artificial intelligence (AI) to speed up counting New Zealand fur seal pups from UAV images (Marchowski, 2021; Hawkins et al., 2023), and the annotated pup count images from this study will be used in ongoing research to train AI models to do this. Given the ease with which New Zealand fur seals can be counted on the barren surface of the Bounties from aerial photos, satellite imagery could also be used to monitor this population in future (Gonçalves, Spitzbart & Lynch, 2020; Larue et al., 2021; Fudala & Bialik, 2022). Such approaches will not be possible for New Zealand fur seals at all colonies in New Zealand given the species’ preference for topographically complex coastlines with caves, overhangs and large boulders to provide shelter (Ryan, Hickling & Wilson, 1997; Bradshaw et al., 1999). That said, there are certain colonies where pups have few hiding places, such as Chalky Island in Fiordland (Chilvers, 2021) or the Kaikōura Peninsula colony (Pers. obs. by A. Hall in 2023), where UAVs or satellite imagery offer reliable and efficient ways to conduct monitoring.

### Comparison with previous New Zealand fur seal estimates

The estimate of 4,252–4,341 pups in 2024 (Table 1) is similar to the estimate of 4,380 in 1994 (Taylor, 1996). However, there are notable differences in the methodologies adopted by the two studies.

First, Taylor’s (1996) data are derived from a combination of monochrome photos taken from an aircraft operating at between ∼366–914 metres altitude, and colour photos taken from ∼90 metres above sea level with a 100 mm lens, for selected areas. By comparison, the UAV used in this study was operating at between 40–60 metres above each island’s highest point and covered the entirety of the Main Group of islands and rocks. Given the lower overall operating height of the UAV compared with Taylor’s (1996) aircraft, and improvements to camera technology in the last 30 years, the images collected here were likely of higher quality than Taylor’s (1996), enabling more reliable pup counts.

There are also differences in the timing of the two studies. It is possible that not all of the 1994 pup cohort had been born when Taylor (1996) conducted his study (7 January, 1994). On mainland New Zealand, New Zealand fur seal pups are typically born between late November and early January (Lalas & Harcourt, 1995; Shaughnessy, Goldsworthy & Libke, 1995), with surveys timed to take place after mid-January to ensure all births have occurred (Chilvers, 2021). On Macquarie Island, fur seal pups are born over a similar time frame, from mid-November to early January (Shaughnessy, Shaughnessy & Fletcher, 1988), while on South Georgia, Antarctic fur seals (*Arctocephalus gazella*) are born between mid-November and late December (Boyd, 1993). As such, while most pups would likely have been born when Taylor (1996) surveyed, it is possible that the entire cohort was not available for counting.

There are also differences between how pup production was calculated between Taylor (1996) and the present study. Taylor (1996) estimated total pup production by first calculating the ratio of new-born pups to older New Zealand fur seals at several locations on the Bounties, and then relating this ratio to the total number of “other seals” counted on the day of surveying. A similar approach was adopted by Taylor (1982) in his previous assessment of the Bounties, when a total population of ∼16,000 New Zealand fur seals was estimated. There are several potential issues with this approach. Firstly, on 7 January, 1994, an unknown number of the “older seals” Taylor (1996) used in the ratio for estimating pup production would have been away from the colony foraging (Sharpies, Mackenzie & Hammond, 2009). The assumption that all pups are on land and available for counting is the primary reason why they, rather than older cohorts, are used to estimate pinniped population sizes (Berkson & De Master, 1985). There are also important demographic unknowns which may affect the reliability of Taylor’s (1996) estimate. For example, had there been unusual (high or low) rates of pup or post-weaner survival (Chambellant et al., 2003; Beauplet et al., 2005) among the cohorts born in the years immediately prior to Taylor’s 1994 (1996) survey, this would have impacted the ratio used in his pup production calculation, despite those individuals not contributing to pup production in 1994.

While the similarities between these results and Taylor’s (1996) could suggest stability in the Bounties’ New Zealand fur seal population, due to the differences in the methodologies, and the fact that the 2024 survey did not cover the entire archipelago, it is not recommended that conclusions are drawn about the trajectory of the Bounties’ population over the last 30 years. Rather, the results presented here should provide a new baseline, and future monitoring should be conducted to map trends in the population.

The only other recent New Zealand fur seal population estimate in the subantarctic region comes from Reef Point on Antipodes Island, where a population of approximately 940 New Zealand fur seals was estimated by Mattern (2024) based on multiplying pup counts derived from UAV surveys in January 2024. From the few mainland New Zealand colonies with recent population estimates, New Zealand fur seal abundance at the Bounties is much larger than at declining colonies on the WCSI (Hall et al., 2026) slightly larger than that of Banks Peninsula, where 13,147–17,675 New Zealand fur seals were estimated in 2024 (Hall, Chilvers & Weir, 2025a), smaller than the∼30,000–33,000 (Hall et al., *unpublished data*) estimated for Kaikōura in 2025 and comparable to that of Lower Fiordland, where ∼14,000–24,000 New Zealand fur seals were estimated in 2021 (Chilvers, 2021). As such, even though this study almost certainly underestimates the true population of New Zealand fur seals at the Bounties, it is likely among the largest and most important recorded in New Zealand to date.

### Study limitations

Given that New Zealand fur seal breeding has previously been recorded on at least one island not included in this study (Funnel Island, the largest island in the Centre Group) this study cannot reliably provide a whole population estimate for the Bounties. Taylor (1982) and Taylor (1996) did not list all of the islands on which New Zealand fur seal breeding was recorded, although he did state that the main breeding grounds were on Penguin, Depot and Proclamation Islands, which was also true in 2024 (Table 1). As such, it cannot be estimated with certainty what proportion of 2024’s pup production may have been missed by only surveying the Main Group. However, Taylor (1996) states that less than 6% of the Bounties’ New Zealand fur seals were recorded on the Centre Group and less than 1% on the East Group in 1980, 1983 and 1994. Similarly, in a previous UAV survey of the Bounties in 2022 (T. Mattern et al., 2022, unpublished data) only ∼3% of the total number of New Zealand fur seals counted were outside the Main Group. The November timing of that 2022 survey means it cannot be used to estimate pup production, and thus population size. Given the continued importance of Penguin, Depot and Proclamation Islands as breeding sites, the much smaller land masses available on most islands of the Centre and East groups and breeding site fidelity among female New Zealand fur seals (Chilvers, 2021), it seems probable that the 2024 survey covered the majority of the important New Zealand fur seal breeding areas on the Bounties. Ideally, future surveys will include the entire archipelago.

Some pups were likely missed through being obscured by features like rocks or overhangs (Taylor, 1996). Previously, obscured vision has led to substantial underestimates in New Zealand fur seal counts using UAVs (Gooday et al., 2018). However, the barren nature of the granite islands and rock stacks that make up the Bounties means that there are far fewer hiding places for pups there compared to many colonies on mainland New Zealand. Taylor (1996) estimated that less than 2% of New Zealand fur seals on the Bounties would be hidden from aerial photography, and this was factored into our calculations. Other factors that may have contributed to pups being missed or incorrectly identified include some blurry images as well as the presence of other species which could be misidentified as pups. To maintain consistency across counts, and thus minimise bias, the same observer, conducting multiple counts, was used for all islands (McIntosh, Holmberg & Dann, 2018). If this study is repeated in future, alternative approaches to counting could include opening the project to citizen scientists (Wood et al., 2021), as has been done with Australian fur seals (Sorrell et al., 2019), or using AI (Marchowski, 2021). Additionally, it would be useful to conduct ground truthing while UAV counts are being undertaken to provide greater certainty over how many pups are likely to be missed.

This study could not fully account for pre-survey mortality, which can be up to 20% during the first 50 days after birth (Mattlin, 1978). While pups that had died immediately prior to surveying may have been unknowingly included in live counts, pups that had died earlier in the pupping season would likely have been invisible due to scavenging or decomposition. This is another reason for believing the results presented here underestimate true pup production for the Bounties in 2024.

The multipliers used in this study should be treated with some caution, given that one (Goldsworthy & Page, 2007) is based on New Zealand fur seal lifetables from a study population in Australia, while the other (Taylor, 1982; Taylor, 1996) is based on vital rate information from other *Arctocephalus* species. Their inclusion here is to provide a ready comparison with other New Zealand fur seal population assessments where the same multipliers have been used (Chilvers, 2021; Hall et al., 2024; Hall, Chilvers & Weir, 2025a), and the estimates provided should be considered coarse, as vital rates can vary spatially and temporally.

Finally, this study is a single year snapshot, after 30 years where no Bounties’ New Zealand fur seal pup surveys were conducted (Taylor, 1996). As has been demonstrated at more consistently surveyed New Zealand fur seal colonies, pup production can vary dramatically from year to year. For example, pup production at Taumaka Island, on the West Coast of New Zealand’s South Island (WCSI) halved between 1998 and 1999, before largely recovering in 2001 (Hall et al., 2026). Similarly, pup production at Ōhau Point halved between 2023 and 2024 in the context of an unusual mortality event (UME) (Hall et al. in prep). As such, the results provided here should ideally represent a starting point for continued monitoring of the Bounties’ pup production to track the population’s future trajectory.

## Conclusions

The pup production estimates derived from this study provide new baselines for the surveyed islands within Bounties, a region in which the New Zealand fur seal population has been understudied, and where the species faces a variety of potential threats. While New Zealand fur seals are not currently classified as endangered, there are examples worldwide of seemingly robust pinniped populations experiencing drastic reductions in their abundance due to factors such as emerging diseases (Ulloa et al., 2023; Campagna et al., 2024) and changes to marine environmental conditions (Roux, 1998; Roux et al., 2002). Therefore, understanding how populations of this key marine sentinel species change through time is important for understanding wider ocean ecosystem health.

##  Supplemental Information

10.7717/peerj.20975/supp-1 Supplemental Information 1 Raw data of pup counts from Bounty Islands in January 2024 Raw data showing the number of New Zealand fur seal/kekeno pups counted from UAV imagery taken in January 2024.

10.7717/peerj.20975/supp-2 Supplemental Information 2 The distribution of New Zealand fur seal pups counted on Spider Island and Seal Rock Each point indicates the position of a New Zealand fur seal pup counted from drone imagery on Spider Island and Seal Rock.

10.7717/peerj.20975/supp-3 Supplemental Information 3 The distribution of New Zealand fur seal pups counted on Depot Island Each point indicates the position of a New Zealand fur seal pup counted from drone imagery on Depot Island.

10.7717/peerj.20975/supp-4 Supplemental Information 4 The distribution of New Zealand fur seal pups counted on Proclamation Island Each point indicates the position of a New Zealand fur seal pup counted from drone imagery on Proclamation Island.

10.7717/peerj.20975/supp-5 Supplemental Information 5 The distribution of New Zealand fur seal pups counted on Ranfurly Island Each point indicates the position of a New Zealand fur seal pup counted from drone imagery on Ranfurly Island.

10.7717/peerj.20975/supp-6 Supplemental Information 6 The distribution of New Zealand fur seal pups counted on Penguin Island Each point indicates the position of a New Zealand fur seal pup counted from drone imagery on Penguin Island.

10.7717/peerj.20975/supp-7 Supplemental Information 7 The distribution of New Zealand fur seal pups counted on Ruatara Island Each point indicates the position of a New Zealand fur seal pup counted from drone imagery on Ruatara Island.

10.7717/peerj.20975/supp-8 Supplemental Information 8 The distribution of New Zealand fur seal pups counted on Lion Island Each point indicates the position of a New Zealand fur seal pup counted from drone imagery on Lion Island.

## Additional Information and Declarations

### Field Study Permissions

The following information was supplied relating to field study approvals (*i.e.*, approving body and any reference numbers): The project was approved by the NZ Department of Conservation. The Ngāi Tahu Research Consultation Committee (Te Komiti Rakahau Ki Tahu) commended this research and deemed it of importance to Māori.

### Data Availability

The following information was supplied regarding data availability: The raw data are included in the Supplementary Files. No code was used in the data analysis.
