# Supplementary figures and images for: First New Zealand fur seal population assessment at the Bounty Islands in 30 years
Source: PeerJ. 2026 Mar 27;14:e20975. doi: 10.7717/peerj.20975 (PMC13034872; doi:10.7717/peerj.20975)

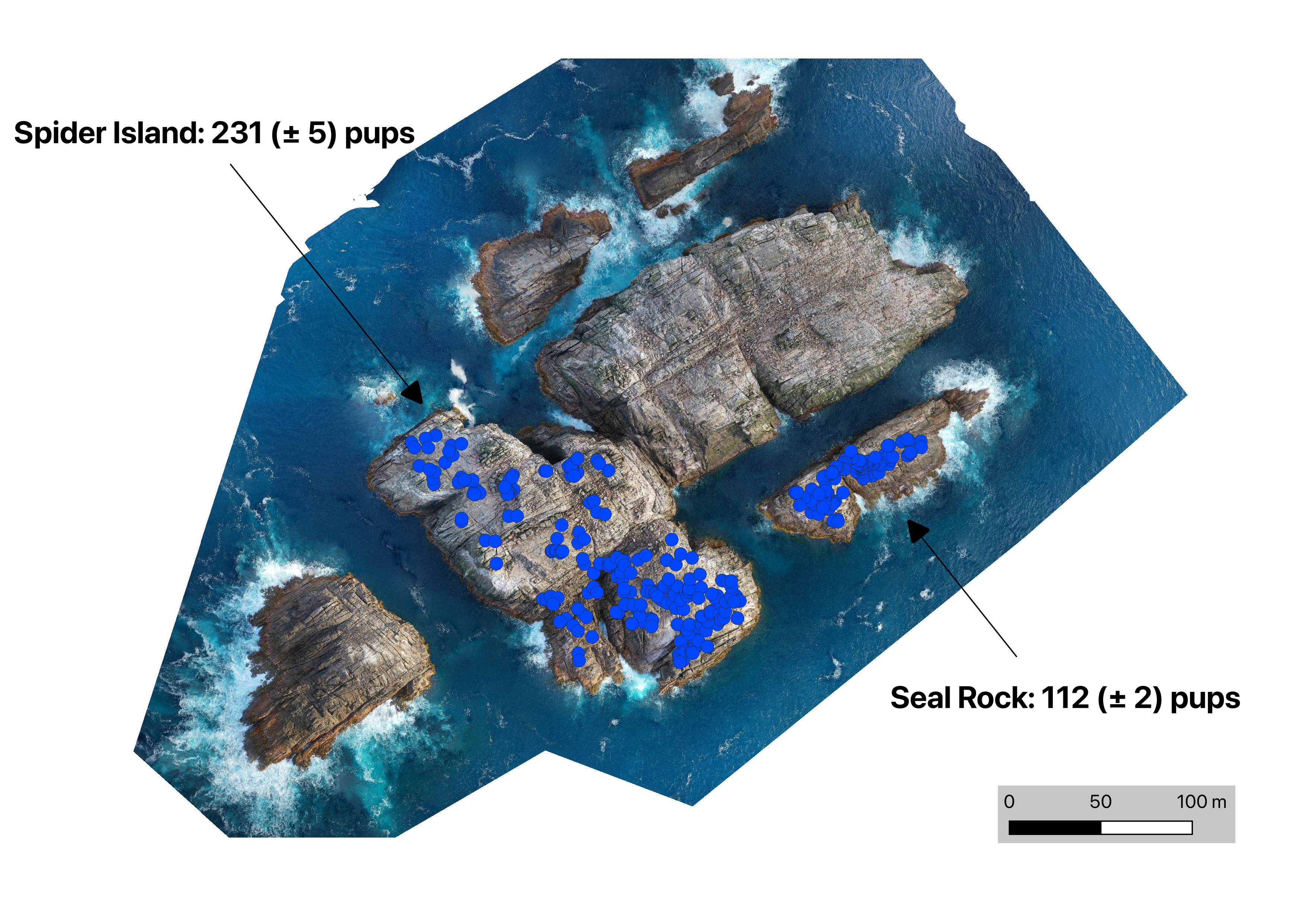

Supplement: Supplemental Information 2 — Each point indicates the position of a New Zealand fur seal pup counted from drone imagery on Spider Island and Seal Rock. [file peerj-14-20975-s002.png]

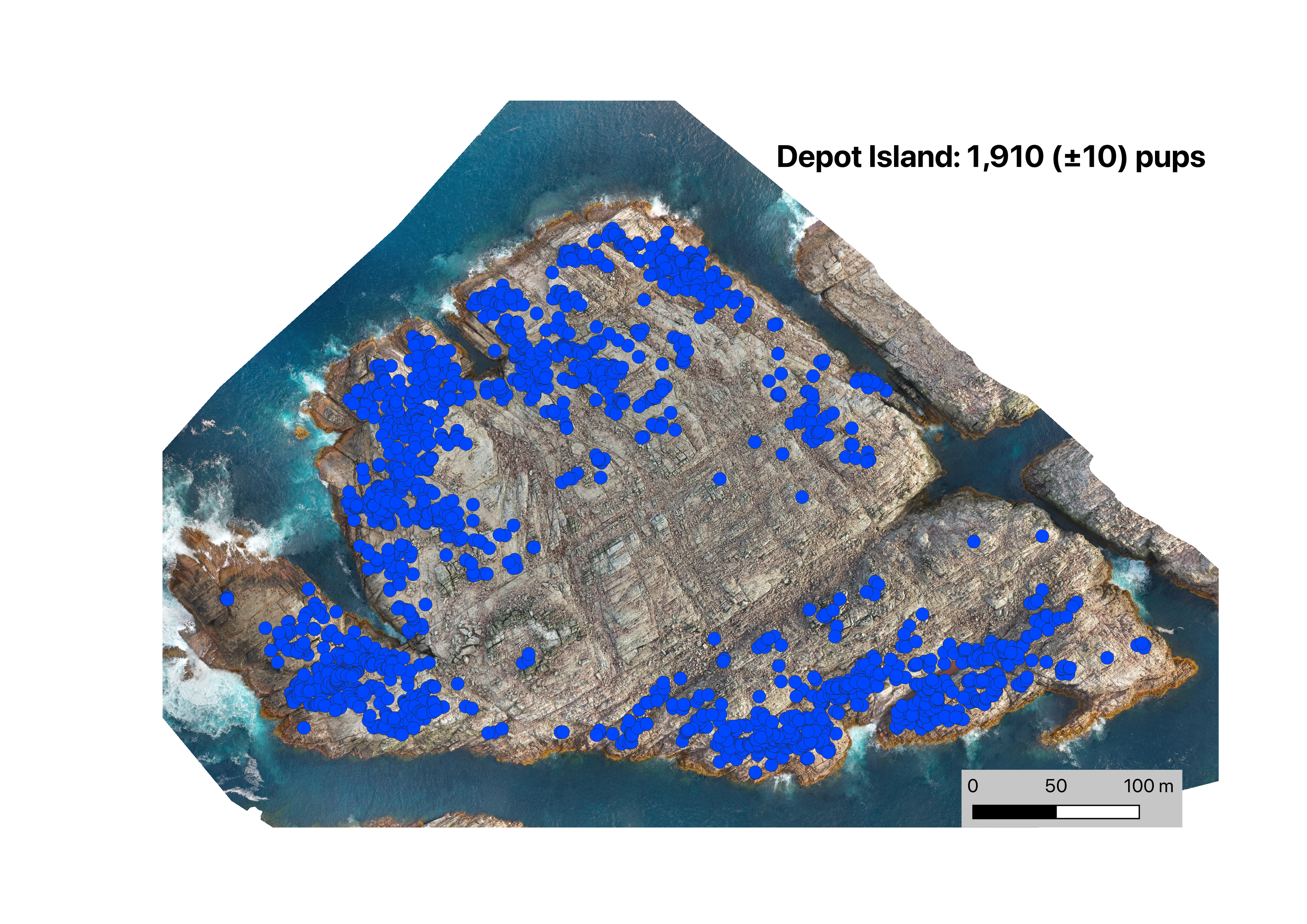

Supplement: Supplemental Information 3 — Each point indicates the position of a New Zealand fur seal pup counted from drone imagery on Depot Island. [file peerj-14-20975-s003.png]

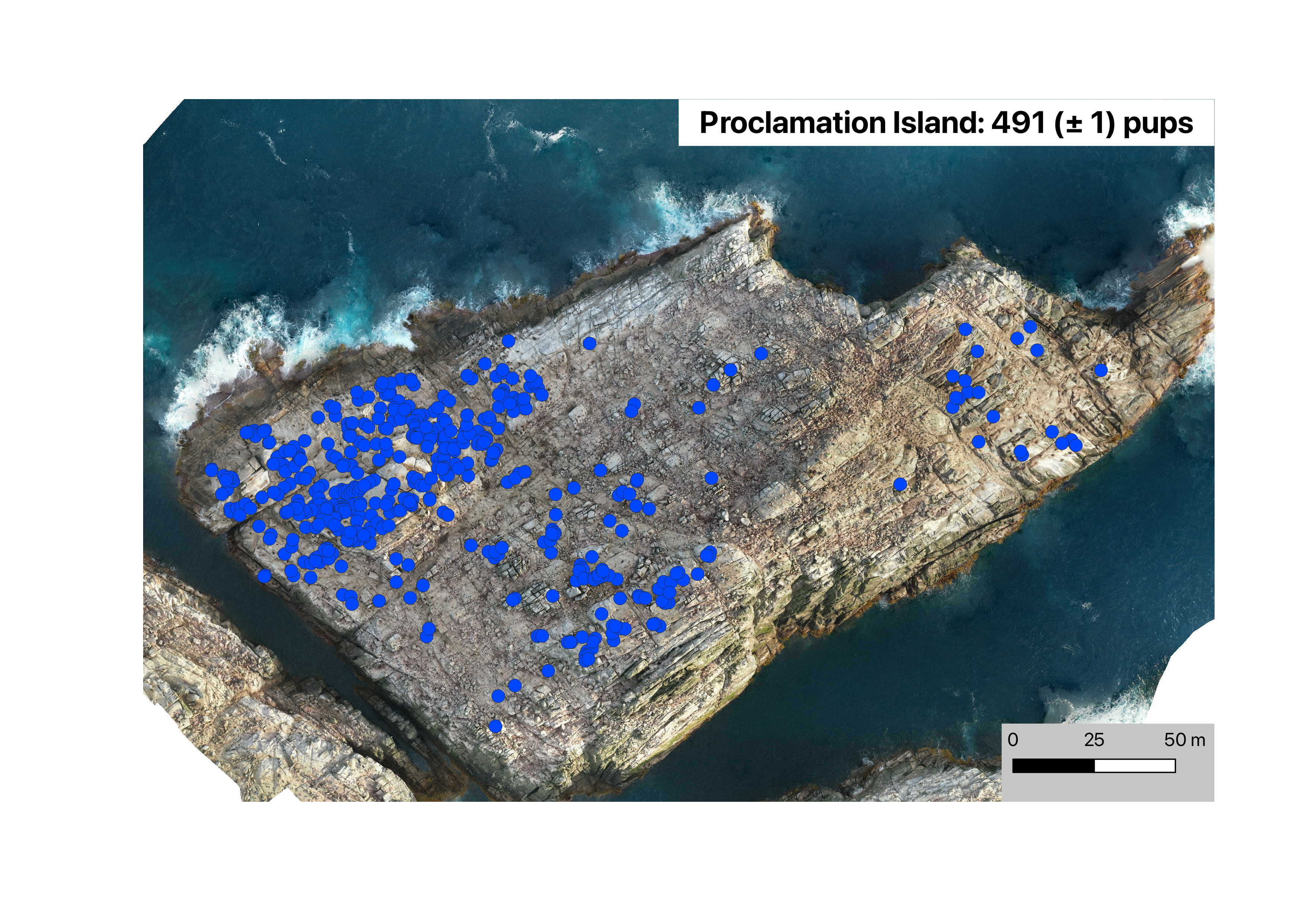

Supplement: Supplemental Information 4 — Each point indicates the position of a New Zealand fur seal pup counted from drone imagery on Proclamation Island. [file peerj-14-20975-s004.png]

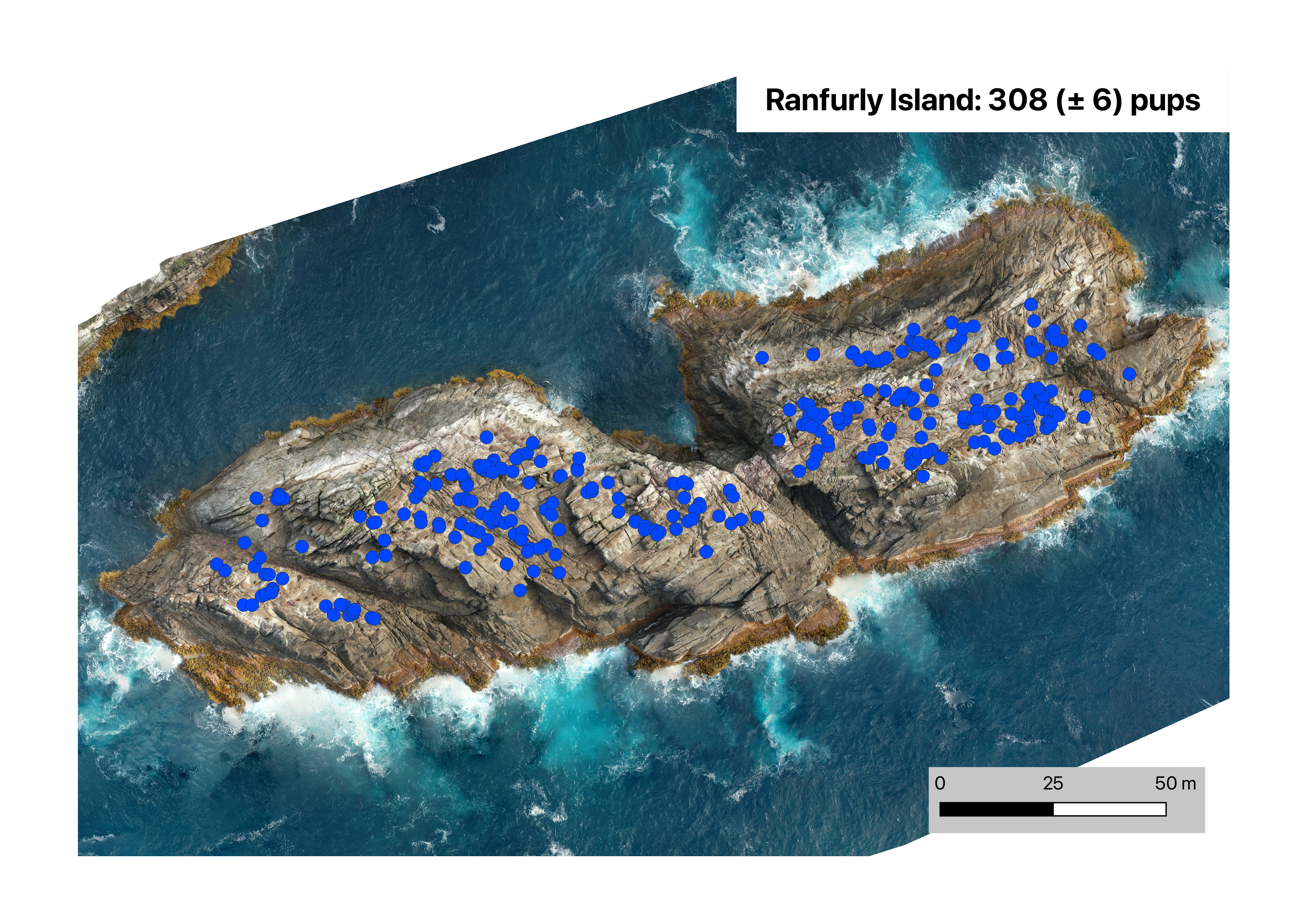

Supplement: Supplemental Information 5 — Each point indicates the position of a New Zealand fur seal pup counted from drone imagery on Ranfurly Island. [file peerj-14-20975-s005.png]

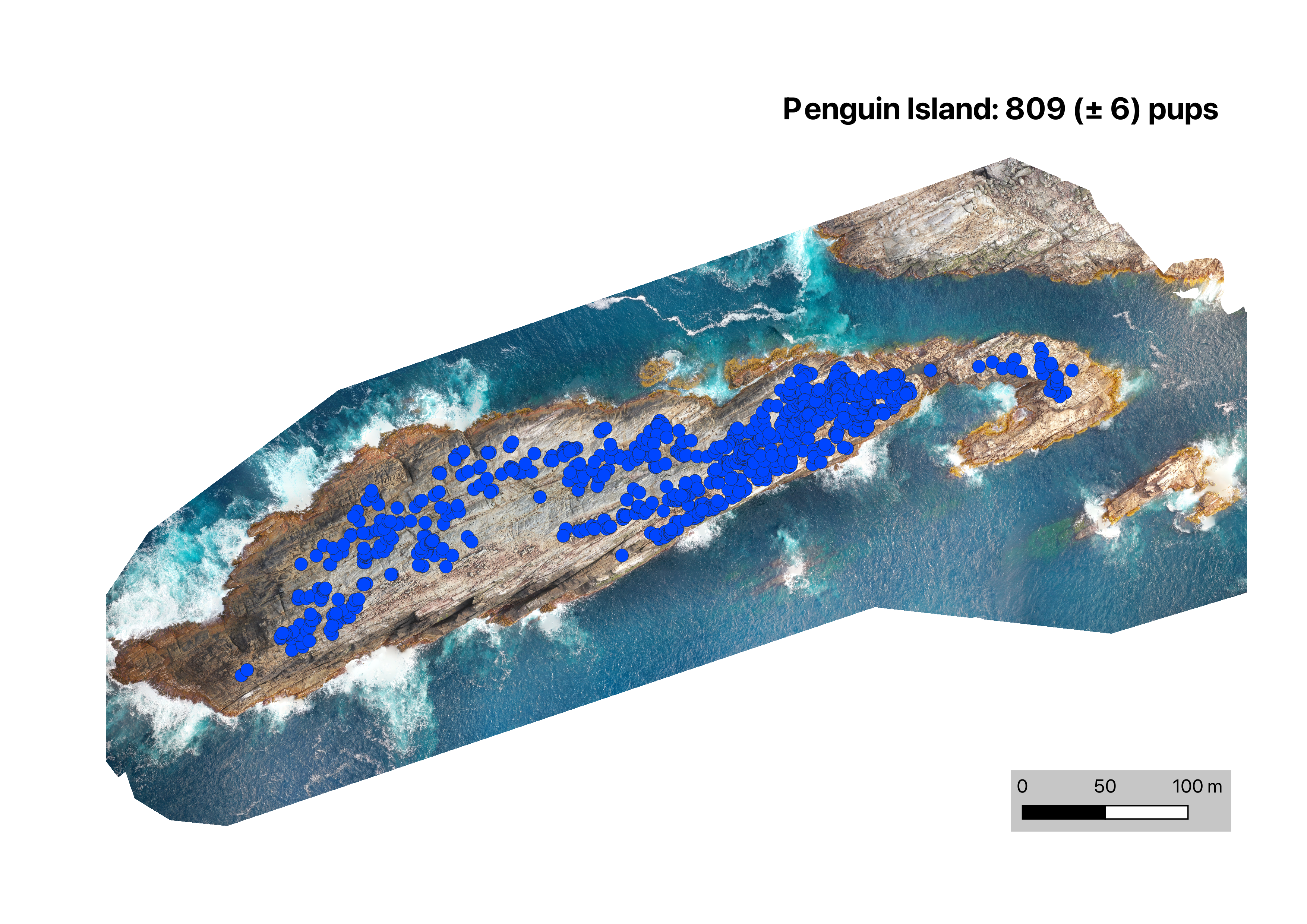

Supplement: Supplemental Information 6 — Each point indicates the position of a New Zealand fur seal pup counted from drone imagery on Penguin Island. [file peerj-14-20975-s006.png]

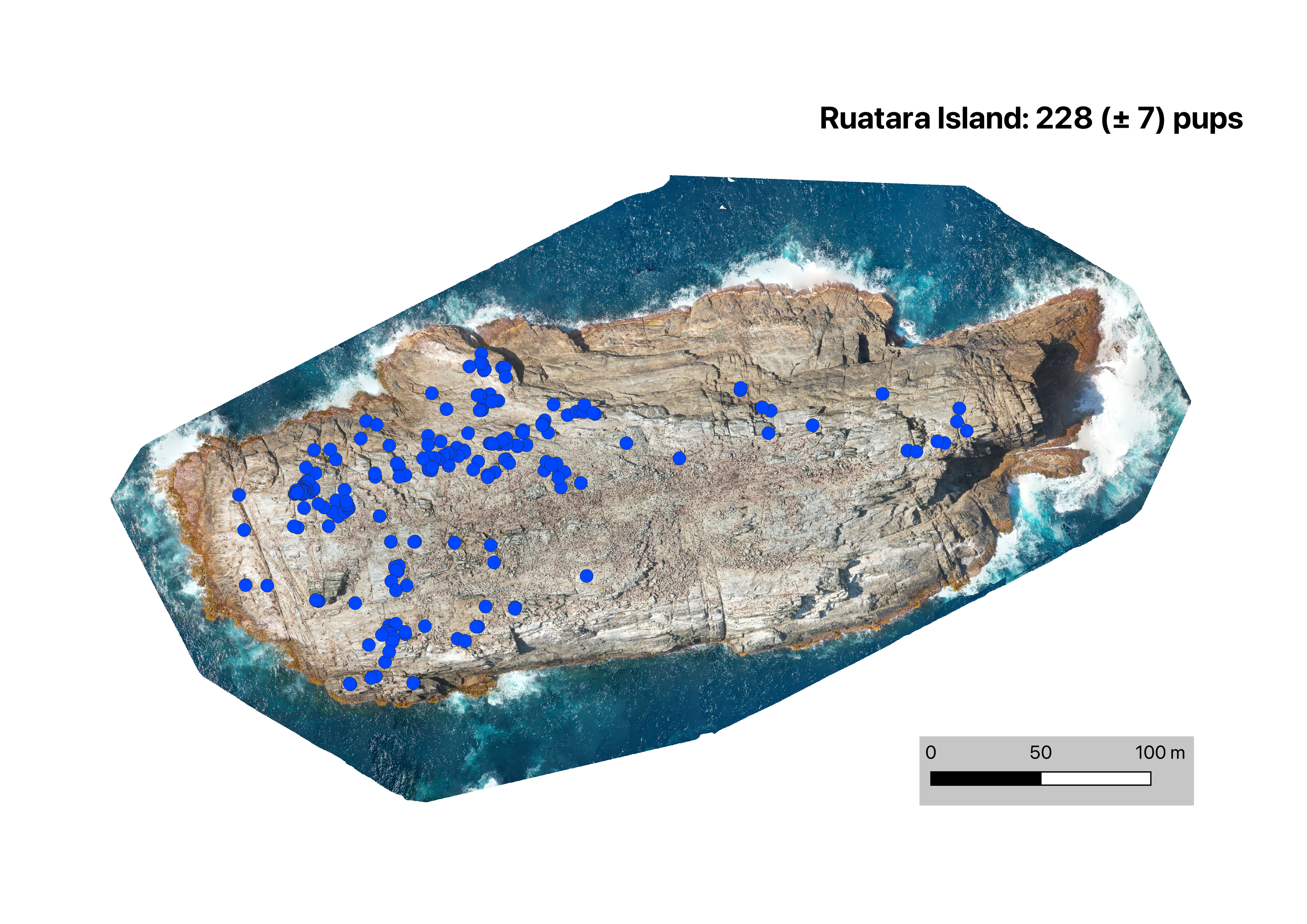

Supplement: Supplemental Information 7 — Each point indicates the position of a New Zealand fur seal pup counted from drone imagery on Ruatara Island. [file peerj-14-20975-s007.png]

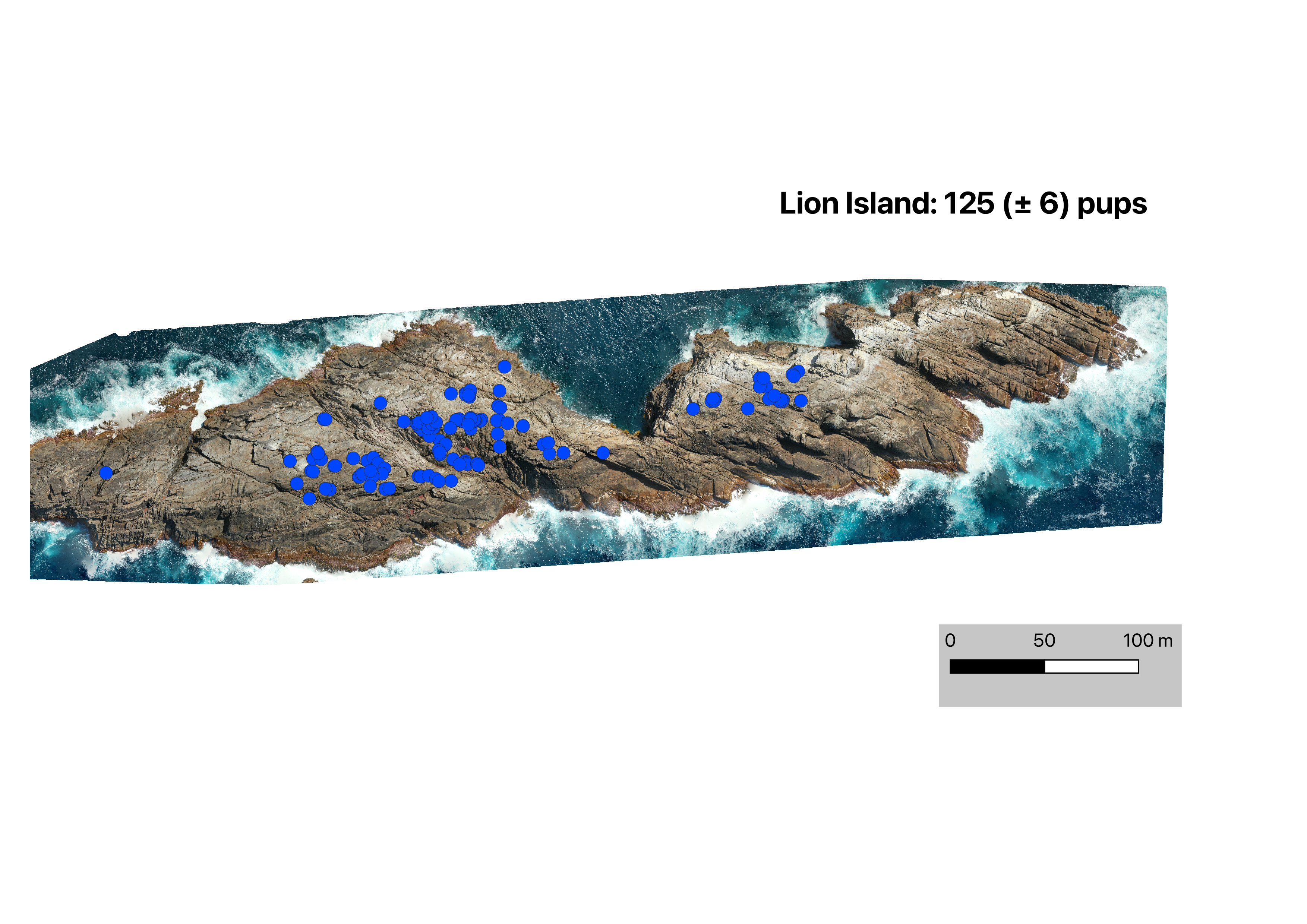

Supplement: Supplemental Information 8 — Each point indicates the position of a New Zealand fur seal pup counted from drone imagery on Lion Island. [file peerj-14-20975-s008.png]
